# Effects of Pressure Rollers with Variable Compliance in the Microfinishing Process Utilizing Abrasive Films

**DOI:** 10.3390/ma17081795

**Published:** 2024-04-13

**Authors:** Katarzyna Tandecka, Wojciech Kacalak, Łukasz Rypina, Maciej Wiliński, Michał Wieczorowski, Thomas G. Mathia

**Affiliations:** 1 Department of Engineering and Informatics Systems, Faculty of Mechanical Engineering and Energy, Koszalin University of Technology, 75-620 Koszalin, Poland; wojciech.kacalak@tu.koszalin.pl (W.K.); lukasz.rypina@tu.koszalin.pl (Ł.R.); 2 Independent Researcher, 75-412 Koszalin, Poland; wilinskimaciej@wp.pl; 3 Faculty of Mechanical Engineering, Institute of Applied Mechanics, Poznan University of Technology, 3 Piotrowo St., 60-965 Poznan, Poland; michal.wieczorowski@put.poznan.pl; 4 Laboratoire de Tribologie et Dynamique des Systemes (LTDS), Ecole Centrale de Lyon, Centre National de la Recherche Scientifique, 69134 Lyon, France; thomas.mathia@ec-lyon.fr

**Keywords:** surface finishing, abrasive film, finishing, abrasion, pressure roller, microfinishing, pressure roller

## Abstract

This article presents a comprehensive investigation into pressure rollers utilized in the microfinishing process, covering aspects such as design, experimental properties, compliance, and finite element simulation. Prototype pressure rollers with unconventional elastomer configurations were designed and analyzed to explore their effectiveness in achieving superior surface finishes. Experimental analysis and finite element simulations were conducted to gain insights into the performance and behavior of these pressure rollers under various loading conditions. This study addresses the validation of constitutive material models used in finite element simulations to ensure accuracy and reliability. The results indicate that the applied material model, validated through experimental analysis, accurately predicts pressure roller behavior. Finite element simulations reveal distinct contact zone patterns and stress distributions across the contact surfaces, highlighting the importance of considering deflection-induced variations in contact behavior. Additionally, the investigation evaluates the effectiveness of different pressure rollers in removing surface irregularities during the microfinishing process. Roller R3 demonstrates the highest efficacy in removing surface peaks, suggesting its potential for achieving superior surface finishes. Overall, this research contributes to the advancement of microfinishing techniques by providing insights into pressure roller design, performance, and behavior, thereby optimizing microfinishing processes to produce high-quality components. The urgency of this study arises from the growing need for exceptional surface finishes in various industrial sectors. With manufacturing industries increasingly pursuing high-precision components boasting flawless surface quality, the significance of microfinishing processes is highlighted.

## 1. Introduction

Microfinishing is a vital process in modern manufacturing industries that contributes to the production of high-precision components with superior surface quality [1,2,3]. Pressure rollers play a crucial role in microfinishing operations, exerting controlled pressure on the workpiece to achieve the desired surface finishes [4]. The effectiveness of microfinishing heavily depends on the design and performance of these pressure rollers [5]. Therefore, understanding their behavior under different conditions is essential for optimizing the microfinishing process and enhancing product quality [6].

**Figure 1:**
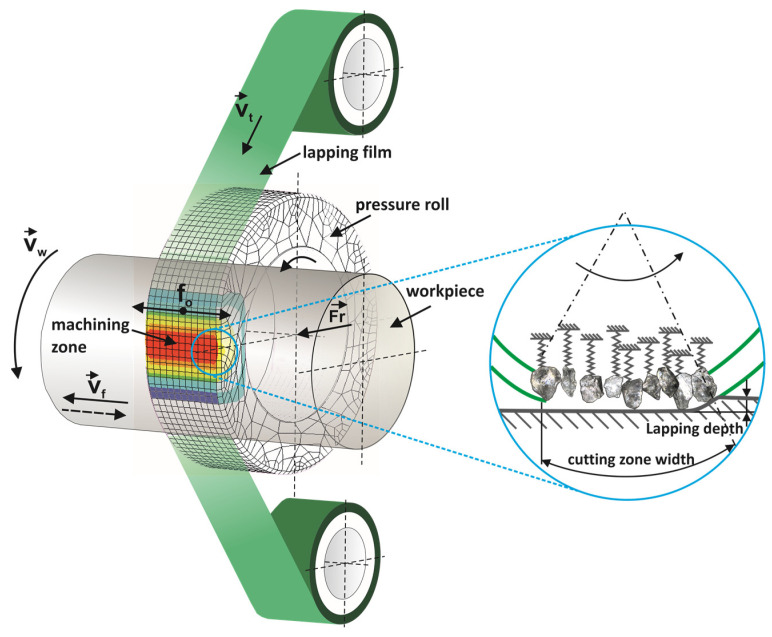
Kinematic diagram of rotary surface finishing using lapping films, where the following quantities are indicated on the diagram: *v_t_*—tool speed, *v_w_*—workpiece speed, *v_f_*—tool feed speed, *f_o_*—tool oscillation frequency, and *F_r_*—the pressure force of the pressing roller [34].

**Figure 2:**
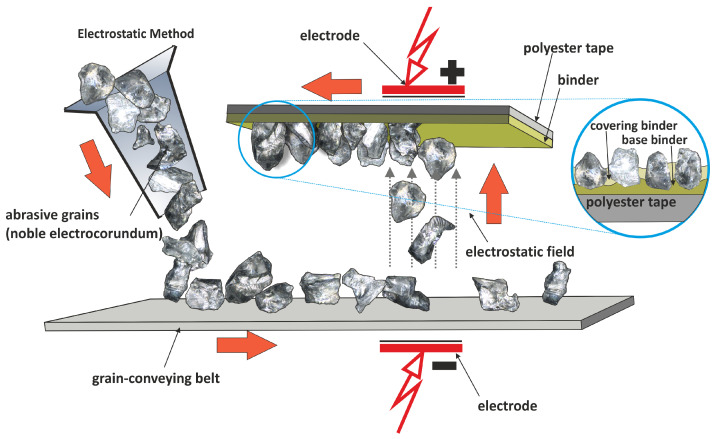
The production scheme in the electrostatic field of microfinishing films [35].

In microfinishing processes, there are two methods utilized for surface treatment: abrasive belt finishing (Figure 1) and grinding with abrasive belts. While both techniques aim to achieve smooth and refined surfaces, they differ significantly in their operational mechanisms and applications. During the microfinishing process, abrasive films typical for microfinishing are utilized [3]. These films are characterized by having abrasive particles (in this study, electrocorundum, nominal size 15 µm) deposited on the backing polyester film in an electrostatic field, ensuring optimal orientation of the abrasive grain for enhanced cutting capabilities (Figure 2). These films are applied to a backing roller and come into direct contact with the workpiece surface [7]. The process usually involves a controlled pressure roller system to ensure consistent pressure distribution across the surface [8]. As the workpiece moves relative to the abrasive film, the abrasive particles abrade the surface, gradually removing material and refining the surface texture [9,10]. The configuration of the abrasive grain is crucial for the effectiveness of the machining process [11,12]. In the case of bonded abrasive tools, the orientation of the grains is crucial for the quality of the resulting surfaces [13]. However, in loose abrasive machining, this process is entirely random [14]. Additionally, in abrasive waterjet machining, the process involves not only abrasives but also the pressure of the water stream [15].

On the other hand, grinding with abrasive belts employs endless belts coated with abrasive particles. These belts are wrapped around rotating drums or rollers and move continuously across the workpiece surface [16]. Unlike abrasive film finishing, where the contact is intermittent, abrasive belts maintain continuous contact with the surface during the entire process [17]. The high-speed movement of the belts facilitates rapid material removal, making this method particularly suitable for rough grinding and shaping operations. Despite these differences, both methods share a similar mechanism of applying pressure to the workpiece [18]. In both abrasive film finishing and grinding with abrasive belts, a pressure system is employed to press the abrasive medium against the workpiece surface [19]. This pressure ensures proper contact between the abrasive particles and the workpiece, facilitating efficient material removal and surface refinement [20].

Using the machining setup depicted in figure number one, two types of finishing tools are employed. Two prominent techniques employed for this purpose are microfinishing utilizing microfinishing film and finishing utilizing lapping film. Microfinishing is a precision machining process designed to improve surface roughness and achieve tight dimensional tolerances [21]. It involves the utilization of abrasive films, particularly microfinishing film, to refine surfaces to micro-inch or even nanometer-level finishes [22]. Microfinishing film comprises a backing material coated with precisely graded abrasive particles (Figure 2) [23]. This abrasive film is adept at removing imperfections such as scratches, tool marks, and surface irregularities, thereby yielding a smooth and uniform surface texture [24]. The process is typically carried out using specialized machinery equipped with rotating spindles and precise control systems to ensure consistency and accuracy [25]. Key advantages of microfinishing with microfinishing film include high precision [26,27], improved performance, and versatility across various materials.

Lapping films, another type of tool used as a disposable tool, differ significantly from microfinishing films in their production process. In this case, abrasive grains are fully embedded within the resin matrix, ensuring that no individual deep scratches are formed, to achieve very precise surface finishes. Typically, in the technological process, microfinishing films serve as a pre-finishing step before smoothing with lapping films, resulting in mirror-like surfaces. The production of lapping films involves a meticulous process where abrasive grains are completely immersed in the resin matrix. Unlike microfinishing films where abrasive particles are adhered to a backing material, lapping films create a uniform layer of abrasives embedded within the resin. This unique manufacturing approach eliminates the risk of individual abrasive particles causing deep scratches on the workpiece surface, ensuring a smoother and more consistent finish [28]. Additionally, the resin matrix offers better control over the distribution of abrasive particles, leading to more precise and uniform material removal during the lapping process [29]. In contrast to microfinishing films, which may leave behind visible scratches or marks due to the nature of their abrasive particles, lapping films deliver exceptionally smooth and pristine surfaces. This makes them particularly suitable for applications where surface quality is critical, such as in optical components or precision engineering parts [30]. Furthermore, the use of lapping films in conjunction with microfinishing films in a sequential processing sequence enhances the overall efficiency and effectiveness of surface finishing operations [31]. By starting with microfinishing to remove larger imperfections and then refining the surface with lapping films, manufacturers can achieve superior results with minimal material removal, reducing waste and improving productivity. Overall, the unique production process and characteristics of lapping films make them indispensable tools for achieving precise and high-quality surface finishes in various industries [32], ranging from semiconductor manufacturing to aerospace engineering. Their ability to produce mirror-like surfaces with minimal surface defects makes them a preferred choice for applications demanding exceptional surface quality and dimensional accuracy [33].

This article presents a comprehensive investigation into pressure rollers utilized in the microfinishing process utilizing abrasive films, covering various aspects such as design, experimental properties, compliance, and finite element simulation. This study focuses on prototype pressure rollers with unconventional elastomer configurations, aiming to explore their effectiveness in achieving superior surface finishes. Through experimental analysis and finite element simulations, this research aims to gain insights into the performance and behavior of these pressure rollers under different loading conditions.

The choice of this topic stems from the increasing demand for high-quality surface finishes in industries such as the automotive [36], aerospace, and precision engineering industries [8]. Achieving the desired surface quality requires advanced microfinishing techniques, where pressure rollers play a pivotal role [37,38]. Despite their importance, there is a lack of comprehensive studies investigating the design and performance of pressure rollers [13], especially those with unconventional configurations [39]. Therefore, this research fills a significant gap in the existing literature and provides valuable insights into improving microfinishing processes.

This investigation also addresses the validation of constitutive material models used in finite element simulations, ensuring the accuracy and reliability of simulation studies. By validating these models through experimental analysis, this study enhances confidence in the simulation results and their applicability to real-world scenarios.

In summary, this article aims to contribute to the advancement of microfinishing techniques by providing a thorough understanding of pressure roller design, performance, and behavior. Through experimental analysis, finite element simulations, and the validation of material models, this research seeks to optimize microfinishing processes, ultimately leading to the production of high-quality components with superior surface finishes.

The urgency of this study stems from the escalating demand for superior surface finishes across diverse industrial sectors. As manufacturing industries increasingly seek high-precision components with impeccable surface quality, the role of microfinishing processes becomes paramount. However, amidst this demand, there exists a pressing need for advancements in microfinishing techniques to meet evolving standards and requirements. Current practices often fall short in delivering the level of precision and quality demanded by modern manufacturing. Therefore, this study embarks on a comprehensive investigation into pressure rollers utilized in microfinishing processes.

## 2. Materials and Methods

As part of the research conducted in this article, prototypes of pressure rollers used in the microfinishing process were designed and fabricated. Subsequently, the experimental properties of these rollers were investigated, followed by the selection of a material model for simulation studies. The final step involved studying the microfinishing process using these rollers. Details of the materials used and the research methods are described below.

### 2.1. Pressure Rollers with Locally Variable Compliance

**Figure 3:**
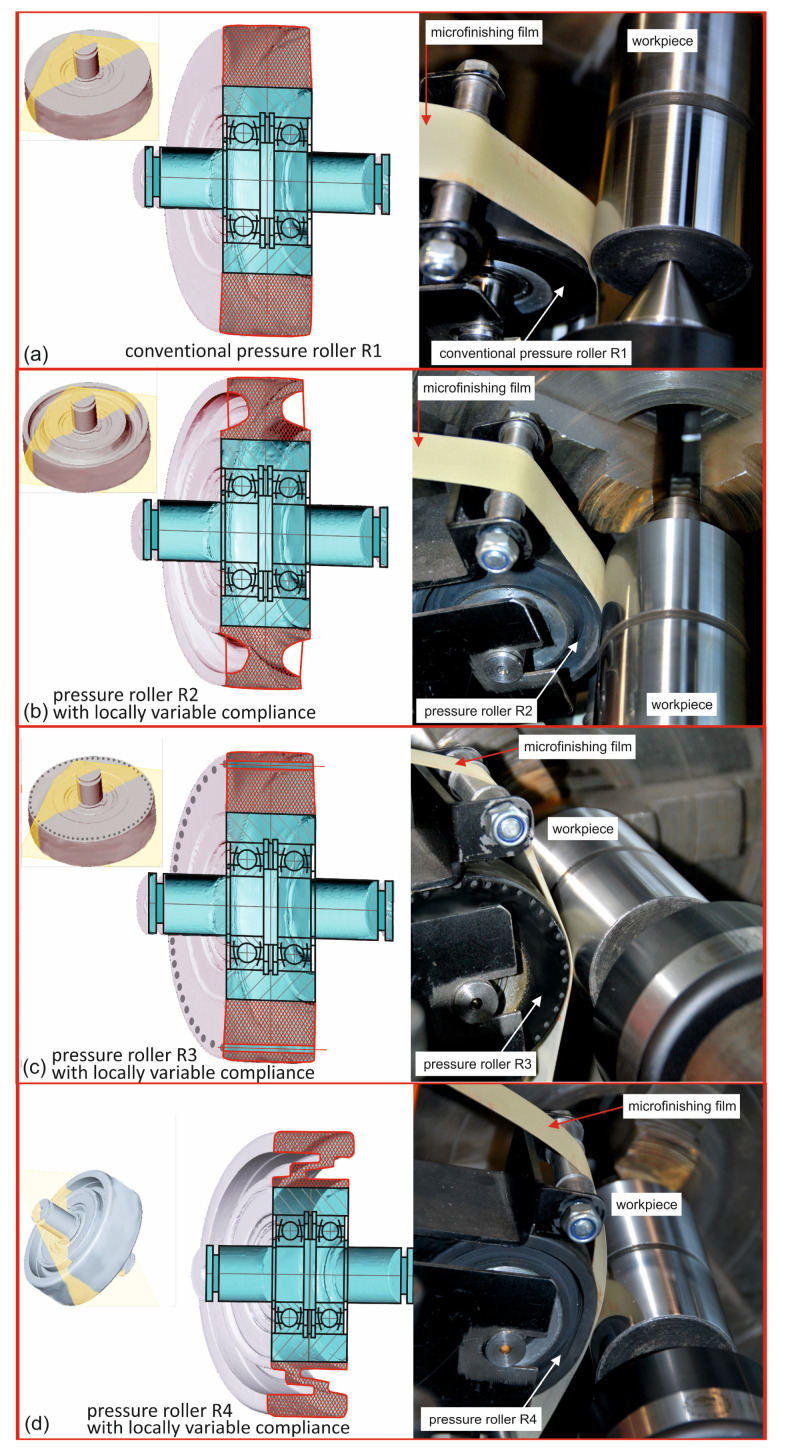
Three-dimensional models, technical drawings, and photos from the microfinishing process of the conventional pressure roller R1 (**a**) as well as the investigated prototypes of pressure rollers: a pressure roller R2 with symmetric lateral cuts in the cross-section of the elastomer layer (**b**), a pressure roller R3 with holes in the elastomer along the cylinder generating line (**c**), and roller R4 with deep, asymmetric cuts in the cross-section of the elastomer layer (**d**).

Four types of pressure rollers were investigated. The first one is the conventional pressure roller, denoted as R1 (Figure 3a), commonly used in typical microfinishing operations with abrasive films [34]. The subsequent pressure rollers are prototypes designed and manufactured at Koszalin University of Technology. These prototype pressure rollers feature unconventional elastomer configurations. The first prototype, designated as R2 (Figure 3b), has symmetric lateral cuts in the cross-section of the elastomer layer. Another prototype, R3, is characterized by holes along the generating line beneath the roller surface (Figure 3c). Lastly, the fourth pressure roller, R4, has bilateral, asymmetric, and deep cuts (Figure 3d). All investigated rollers utilize the same type of elastomer with a hardness of 80 degrees on the Shore A scale.

### 2.2. Research on the Compliance of Pressure Rollers

**Figure 4:**
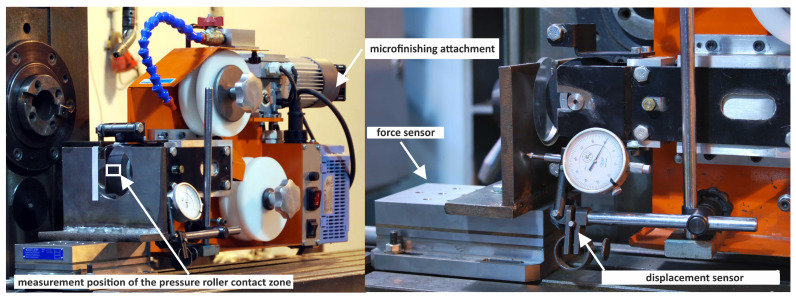
Research setup for the pressure roller contact zone.

An integral part of the research on the prototype pressure rollers was determining their compliance and the surface contact area with a flat surface as a function of the pressing force. For this purpose, a research setup was constructed (Figure 4). GW–1 microfinishing attachment external cylindrical surfaces were used for the experiments, and a transparent pressing surface was designed to enable the determination of the contact surface area of the rollers with the flat surface. The roller pressing against the machined surface in the GW–1 microfinishing attachment was performed pneumatically. The transparent pressing element was mounted on a piezoelectric force sensor 9257B from KISTLER (Winterthur, Switzerland), which was connected to a multi-channel signal amplifier 5070A 12100 from KISTLER, a PC equipped with a measurement card 2855A4 from KISTLER. After mounting the displacement sensor, the relationship between the deflection of the tested prototype pressure rollers and the normal force was determined.

### 2.3. Modeling Stress and Material Displacement in Pressure Rollers Using the Finite Element Method 

The aim of the finite element method-based simulation of roller deformations was to analyze the deformation and stress values in the elastomer layer of the pressure rollers in an Ansys Workbench environment. The input parameters for the simulation were the deflection value f [mm] and material constants. The output parameters included material displacement of the roller and Huber–Mises equivalent stresses. The constitutive material model used refers to the second Piola–Kirchhoff stress tensor. The Blatz-Ko equation, describing a hyperelastic rubber model, was employed in the analysis [40,41]. The general form of the Blatz-Ko equation is as follows (1):

(1) $$S_{ij}=G\left[\frac{1}{V}C_{ij}-V^{\left(\frac{1}{1-2v}\right)}{∆}_{ij}\right]$$

where the variables stand for the following:

G—transverse elasticity modulus;
V—relative volume;
C_ij_—Cauchy–Green deformation tensor;
Δ_ij_—Kronecker delta.

The application of the contact phenomenon model, commonly known as the ‘penalty function model’, was employed in this study. In this model, the determination of the normal force acting in the contact pair is based on relationship (2):

(2) $$F_{n}=\xi u_{n}H\left({-u}_{n}\right);\;\xi =\frac{1}{K}$$

where H is the Heaviside function, K is the penalty function coefficient, and u_n_ is the relative displacement in the normal direction of the bodies in contact.

The material constants of the workpiece material and the material constants used in the Blatz-Ko constitutive model are presented in Table 1.

The numerical model was built using the Ansys Workbench application. The following steps were taken to construct the model:

- A geometric model of the pressure roller–workpiece system was created.
- The calculation methods and discretization scale were determined.
- The materials used in the simulation, as well as the strength models, were defined.
- The type of contact between the tool and the workpiece material was selected.
- The boundary condition parameters were defined.

**Figure 5:**
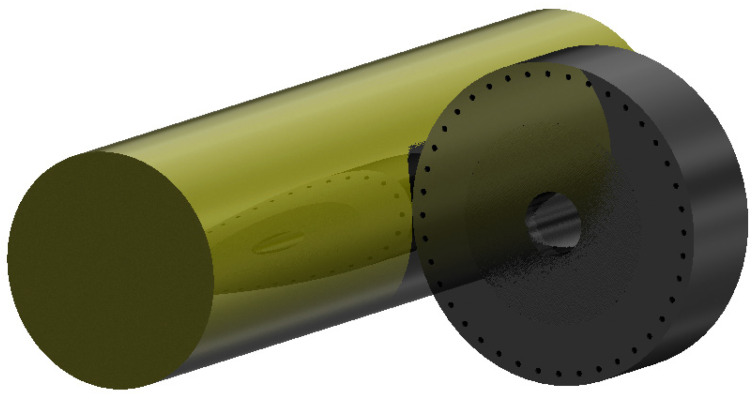
Example of the investigated object’s geometry.

The solid 3D geometric model of the roller–shaft system was prepared using the Inventor environment (Figure 5).

**Figure 6:**
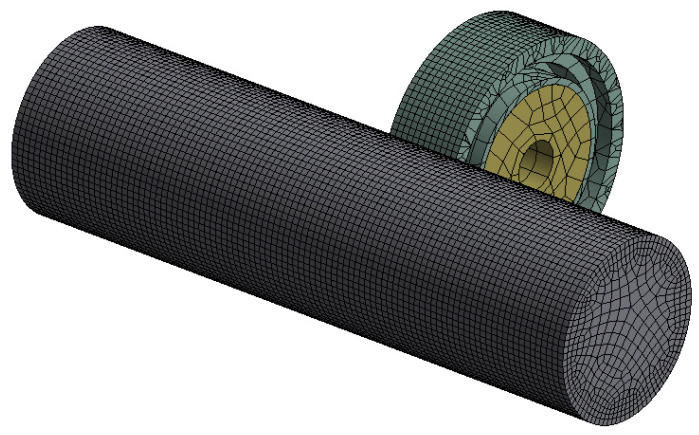
Example of the computer model under investigation.

The simulation was conducted for the case of the spatial deformation state. Solving the posed problem of determining stresses and material displacements of the pressure roller was performed using the implicit integration method. The machined shaft was modeled as a deformable body, while the pressure roller was modeled as a hyperelastic element. The object was discretized with approximately 70,000 eight-node solid elements (Figure 6). Due to the analyzed geometry, the SOLID 186 finite element type with a nonlinear shape function was chosen, which ensured good mesh fit to the geometry, particularly on radii. Translational and rotational degrees of freedom were constrained on the lateral surfaces of the shaft. Subsequently, displacement boundary conditions were applied to the roller, with a displacement of a = 2 mm.

### 2.4. Microfinishing Process Research

For the research on the finishing process, the GW–1 attachment was utilized (Figure 4), enabling interchangeable use of films with widths of 1/2″, 1″, or 2″. The microfinishing film feed speed was vf = 0…90 (max 500) mm/min; the oscillation frequency was f = 0…500 cycles/min, with an oscillation amplitude A = 2.5 mm. The pressure force range of the roller was Fn = 10…90 N, actuated by a pneumatic actuator powered from a network with a pressure of 0.6 MPa.

**Figure 7:**
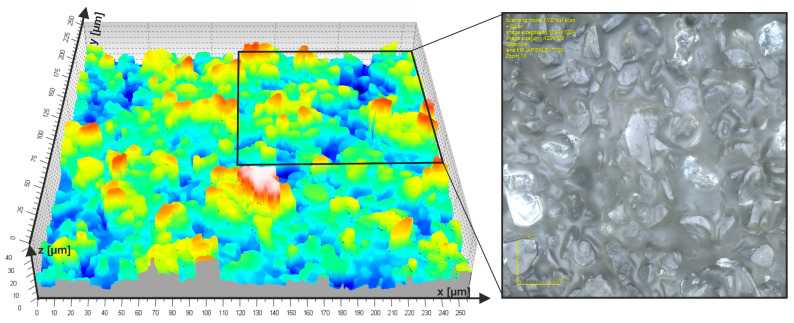
The topography image of the microfinishing film surface with a nominal grain size of 15 µm and an optical photograph of the tool surface measuring 129 × 129 μm, taken using the Olympus OLS4000 (Tokyo, Japan) confocal microscope.

The workpiece processed was a steel shaft made of 41Cr4 (40H) steel, hardened to a hardness of 60 HRC. Steel Mechanical Property: Tensile Strength 1000 MPa, Yield Strength 800 MPa, Elongation 9%. The machining was carried out for 126 s with a workpiece feed speed of 35 m/min, using pressure rollers with an elastomer hardness of 80°Sh A (Table 2). The microfinishing feed speed was 160 mm/min, and the pressure force was 60 N; the nominal abrasive grain size on the microfinishing film was 15 μm (Figure 7). Surface topography measurements were conducted using a TalySurf CCI 6000 measurement system (Taylor Hobson: Leicester, England) every 6 s, i.e., every two passes of the tool over the entire surface of the workpiece. Surface measurements after finishing were performed at three points located every 120° around the circumference of the sample, and the obtained results were averaged for analysis.

## 3. Results and Discussion

### 3.1. Experimental Study of Pressure Roller Deflection

**Figure 8:**
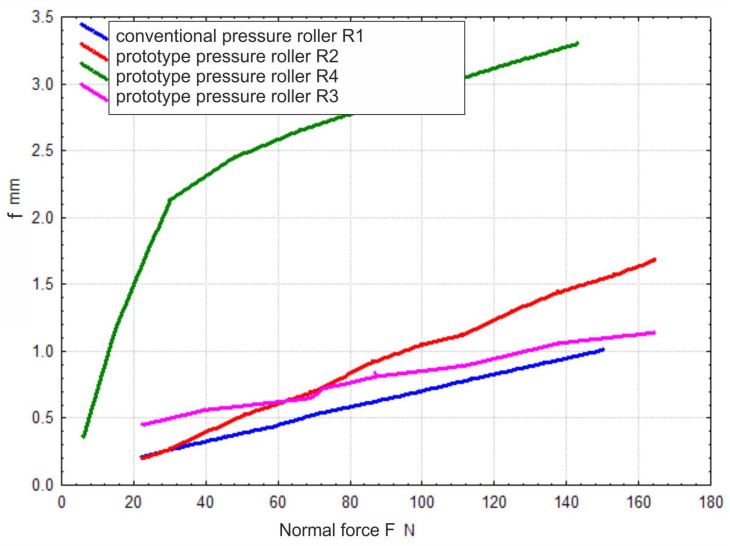
The graph of the deflection of pressure rollers as a function of the applied normal force.

After mounting the displacement sensor, the relationship between the deflection of the tested pressure roller prototypes and the normal force was determined (Figure 4). It was observed that for a compression force of 140 N, the deflection value *f* of the modified pressure roller R2 (Figure 8) was 54% higher compared to the deflection value of the conventional pressure roller R1, which was 0.95 mm. Through the modification of the R2 roller design, a roller with higher flexibility compared to conventional solutions was achieved. Furthermore, the increased deflection of R2 leads to a broader contact area between the pressure roller and the workpiece surface, resulting in a wider machining zone width. This expanded contact area may facilitate more uniform material removal and surface finishing across the workpiece, contributing to improved machining efficiency and surface quality. Until reaching a compression force of 60 N, roller R3 exhibited high compliance; however, once this threshold was exceeded, the compliance value of the roller approached that of the conventional roller. Roller R3 was highly compliant on the outer periphery due to discontinuities in the elastomer material. However, once this threshold was exceeded, the compliance of the roller became similar to that of the conventional roller R1. However, a threefold higher deflection was recorded for roller R4 compared to the conventional roller R1, ensuring excellent conformity to the curvature of the machined workpiece. 

### 3.2. Validation of the Material Model 

**Figure 9:**
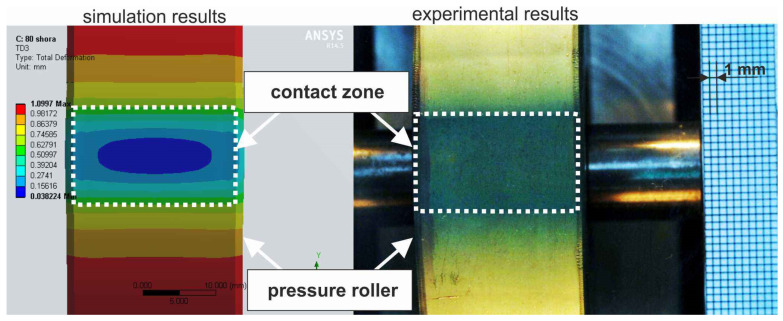
Contact zone after simulation and experiment for a deflection value of 1.1 mm of pressure roller R1.

The applied constitutive material model refers to the second Piola–Kirchhoff stress tensor [42,43]. In the analysis, the Blatz-Ko equation, which describes the hyperelastic model of rubber, was utilized [44]. To ensure the correctness of the conducted analysis, the material model was validated. Compression of the conventional pressure roller R1 against a flat surface was simulated, and experiments were conducted on the test rig presented in Figure 4, utilizing a vision system for data acquisition, made possible by a transparent visor onto which the pressure roller was pressed. The width of the contact zone was examined as a function of the deflection, and comparisons were made with simulation results (Figure 9).

**Figure 10:**
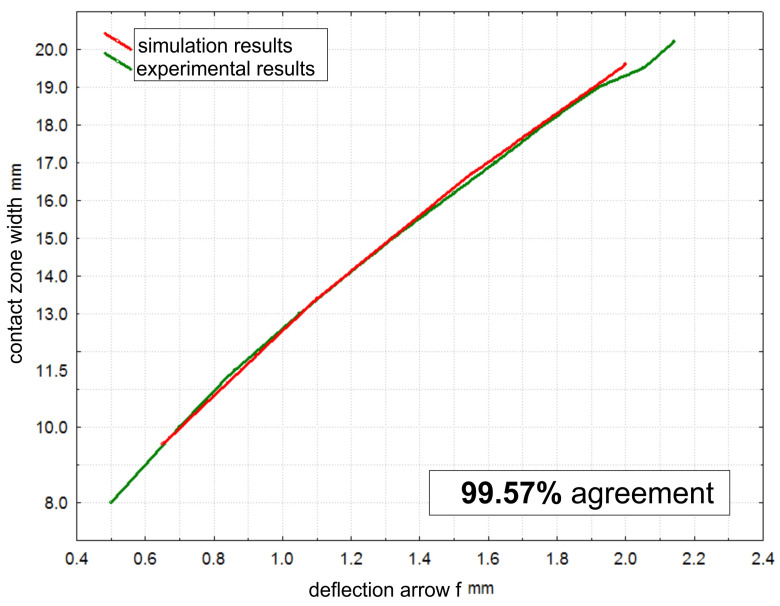
The contact zone width as a function of deflection during the validation process of the material model applied in the simulation using the finite element method of pressure roller R1.

The agreement of the results was calculated at each measurement point (Figure 10), determining it at an overall level of 99.57%, allowing us to conclude that the applied material model is suitable for further simulation studies.

### 3.3. Dynamic Compression Testing of Rollers Using Finite Element Method Simulation

**Figure 11:**
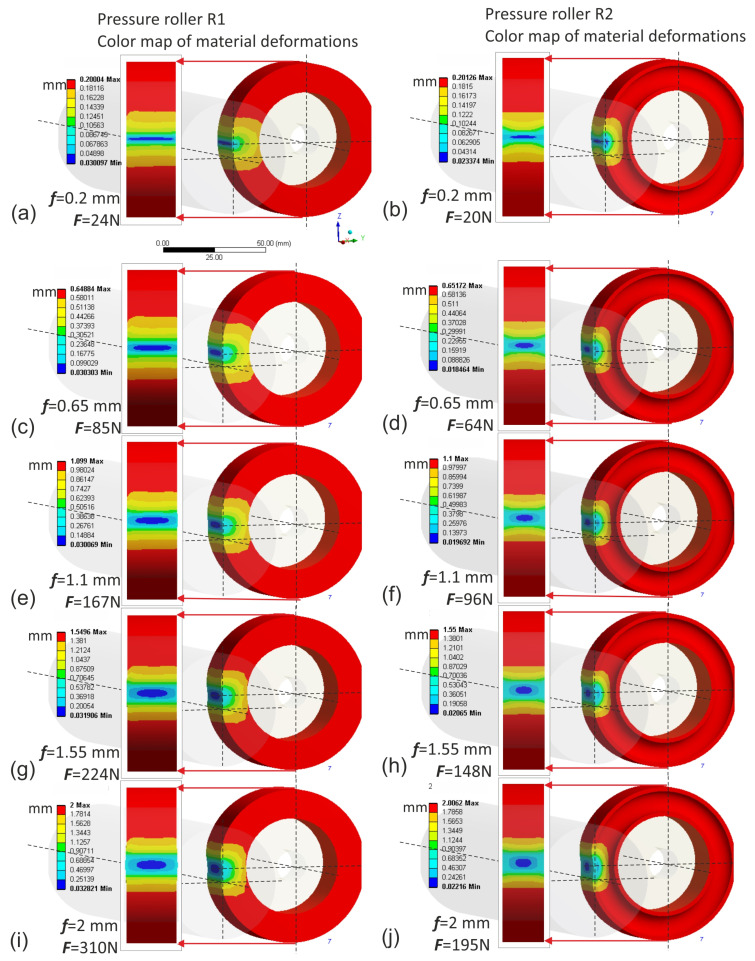
Displacements of the elastomer layer of the conventional pressure roller R1 for successive values of deflection arrow f, 0.2 mm (**a**), 0.65 mm (**c**), 1.1 mm (**e**), 1.55 mm (**g**), 2 mm (**i**), and for the prototype pressure roller R2 for successive values of deflection arrow f, 0.2 mm (**b**), 0.65 mm (**d**), 1.1 mm (**f**), 1.55 mm (**h**), 2 mm (**j**).

**Figure 12:**
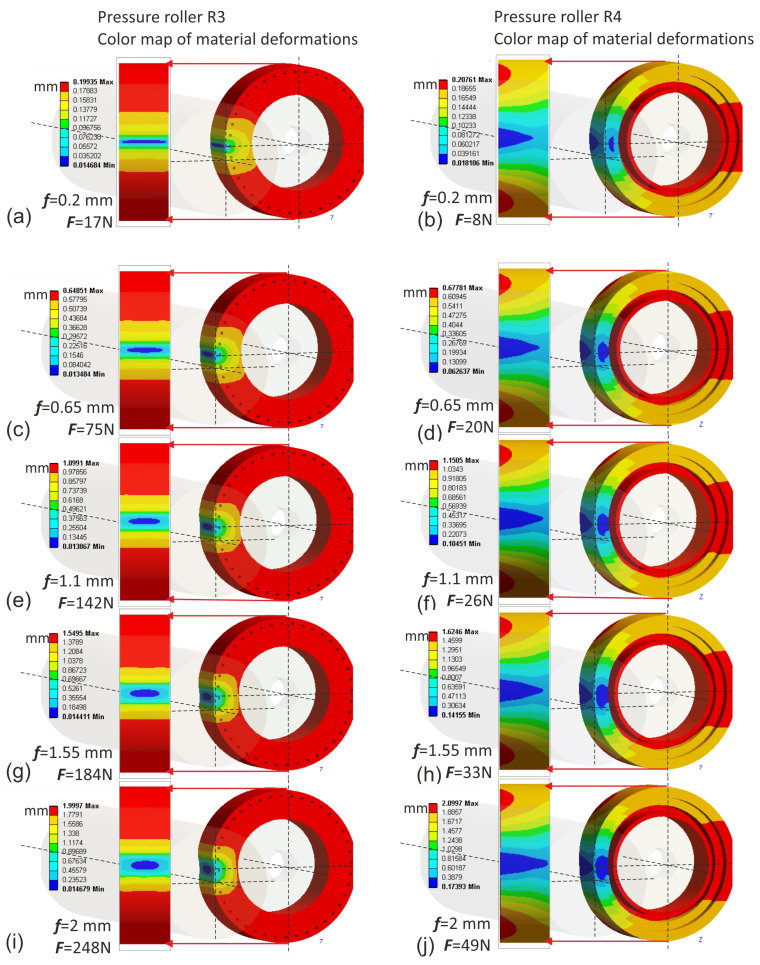
Displacements of the elastomer layer of the prototype pressure roller R3 for successive values of deflection arrow f, 0.2 mm (**a**), 0.65 mm (**c**), 1.1 mm (**e**), 1.55 mm (**g**), 2 mm (**i**), and for the prototype pressure roller R4 for successive values of deflection arrow f, 0.2 mm (**b**), 0.65 mm (**d**), 1.1 mm (**f**), 1.55 mm (**h**), 2 mm (**j**).

**Figure 13:**
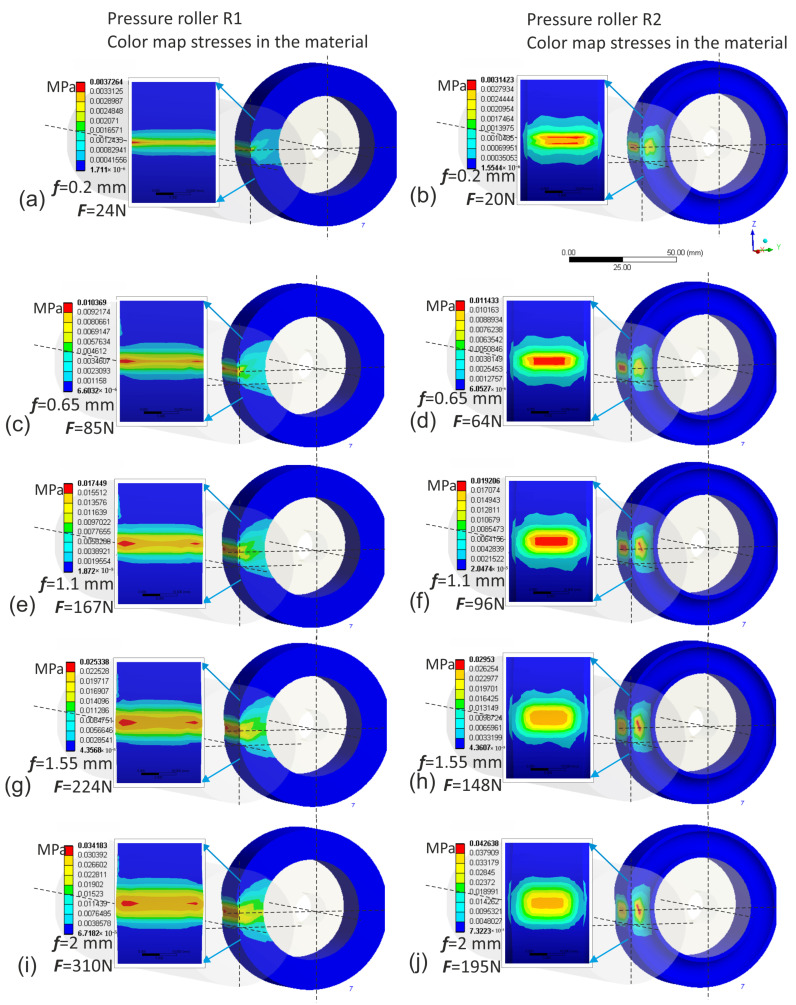
Stresses in the material of the conventional pressure roller R1 for successive values of deflection arrow f, 0.2 mm (**a**), 0.65 mm (**c**), 1.1 mm (**e**), 1.55 mm (**g**), 2 mm (**i**), and for the prototype pressure roller R2 for successive values of deflection arrow f, 0.2 mm (**b**), 0.65 mm (**d**), 1.1 mm (**f**), 1.55 mm (**h**), 2 mm (**j**).

**Figure 14:**
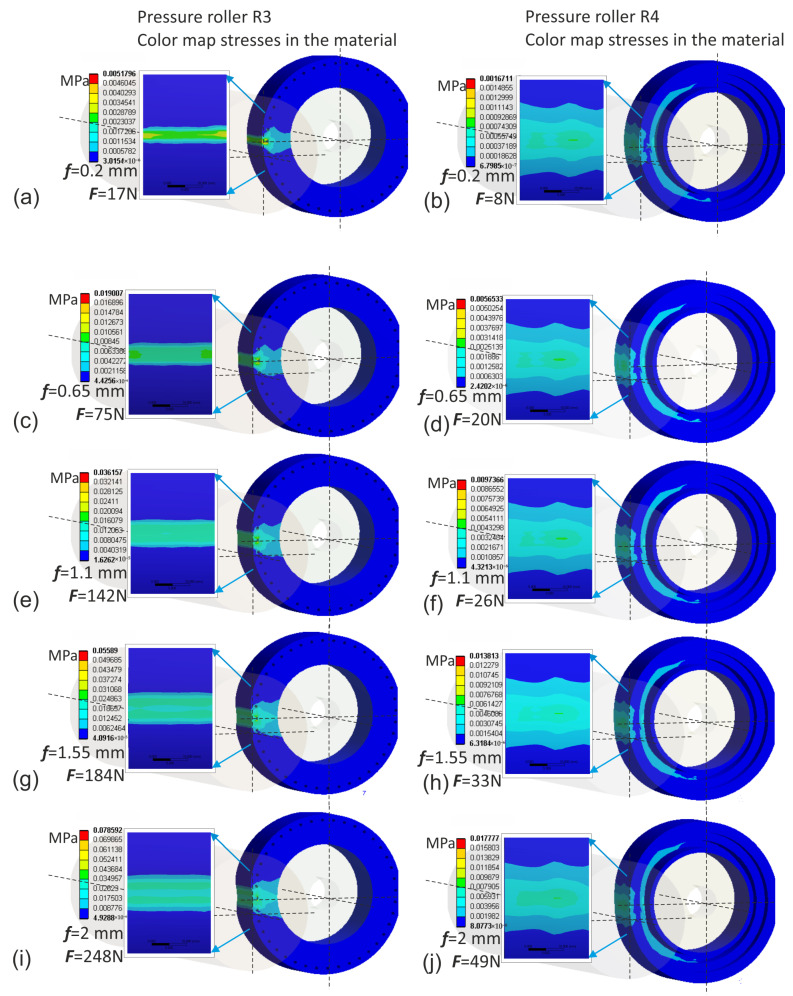
Stresses in the material of the prototype pressure roller R3 for successive values of deflection arrow f, 0.2 mm (**a**), 0.65 mm (**c**), 1.1 mm (**e**), 1.55 mm (**g**), 2 mm (**i**), and for the prototype pressure roller R4 for successive values of deflection arrow f, 0.2 mm (**b**), 0.65 mm (**d**), 1.1 mm (**f**), 1.55 mm (**h**), 2 mm (**j**).

The finite element simulation was conducted using the Ansys environment. The input data for the simulation were deflection values ranging from 0.2 to 2 mm. Naturally, for these same deflection values of individual rollers, different normal force values were obtained when pressing the roller against the workpiece. The highest normal force values were observed in the case of the conventional pressure roller R1, where for a deflection arrow equal to 2 mm, it reached up to 310 N, which is impractically high (Figure 11). In contrast, for prototype roller R4, for the same deflection arrow of 2 mm, only 49 N was observed (Figure 12), indicating that roller R4 operates least aggressively on the workpiece, with the widest contact zone and the highest compliance, thus offering better adaptation to the shape of the machined surface. Prototype roller R3 exhibits a very similar material deformation pattern compared to the conventional roller (Figure 12), while showing higher compliance due to the applied discontinuities, resulting in lower normal force values, even by 20% (Figure 12j) compared to conventional roller R1. The prototype roller R2 generates a very interesting contact zone (Figure 11), where the highest pressures occur in the middle zone of the roller, which may have a positive effect on mitigating the deterioration of surface quality when the tool enters the material during microfinishing with the applied feed. This conclusion is also confirmed by the stress analysis in the pressure roller material (Figure 13). The highest stresses occur at the edges of the conventional roller, reaching up to 0.034 MPa, while for the prototype roller R2, this stress range is observed in the middle machining zone, which is a positive phenomenon. Despite having a very similar machining zone, the prototype roller R3 exhibits a more even distribution of stresses in the material in the direction of the tool feed (Figure 14), which may also have a positive impact on the machining results. The lowest stress values in the material were observed in the case of the prototype roller R4, where the maximum stresses in the outer part of the roller do not exceed 0.0079 MPa.

### 3.4. Experimental Research

A micromachining process utilizing abrasive film with electrocorundum grains deposited on the carrier surface in an electrostatic field was conducted. An abrasive film with a nominal grain size of 15 μm was selected, and all surfaces were smoothed using the same tool from a single production roller. The machined surface was examined at intervals during micromachining. The workpiece was a conventional roller chosen to match the shape of the machined object, so as not to discredit the conventional pressure roller R1, which was dedicated to such a shape of the machined object. In order to compare the micromachining effects from each tested surface, height parameters were determined to evaluate surface roughness according to the ISO 25178 standard [45]:

- Sp—maximum height of peaks;
- Sv—maximum height of valleys;
- Sz—maximum height of the surface;
- Sa—arithmetical mean height of the surface.

**Figure 15:**
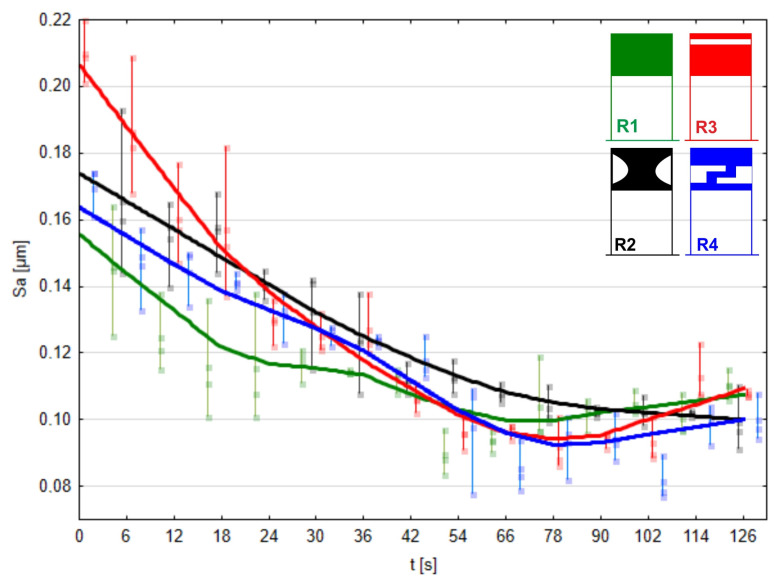
Arithmetical mean height of the machined surface using the conventional pressure roller R1 and prototype rollers R2, R3, and R4 as a function of processing time.

**Figure 16:**
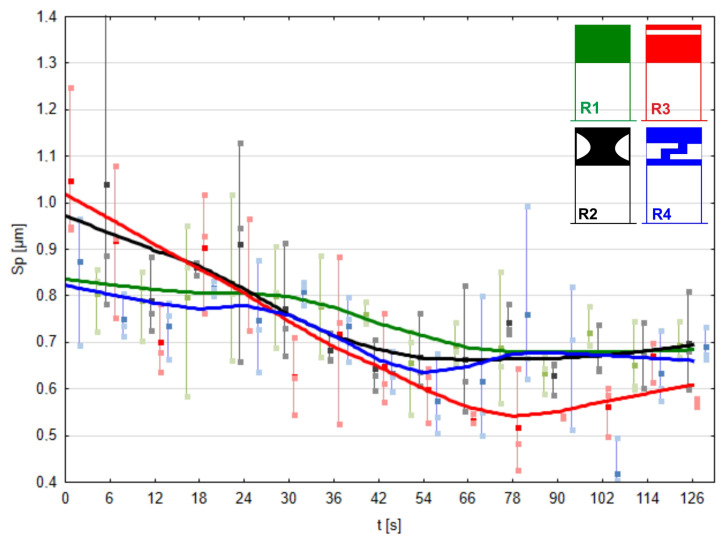
The maximum height of peaks on the machined surface as a function of processing time using the conventional pressure roller R1 and prototype rollers R2, R3, and R4.

**Figure 17:**
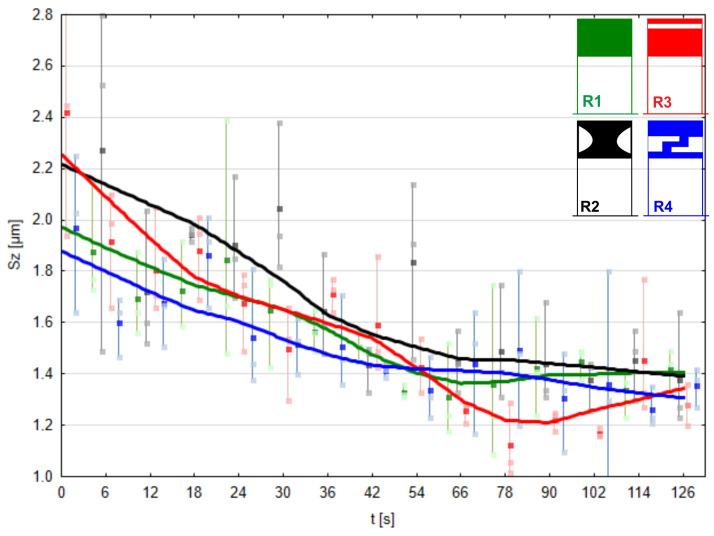
Maximum height of the machined surface using the conventional pressure roller R1 and prototype rollers R2, R3, and R4 as a function of processing time.

**Figure 18:**
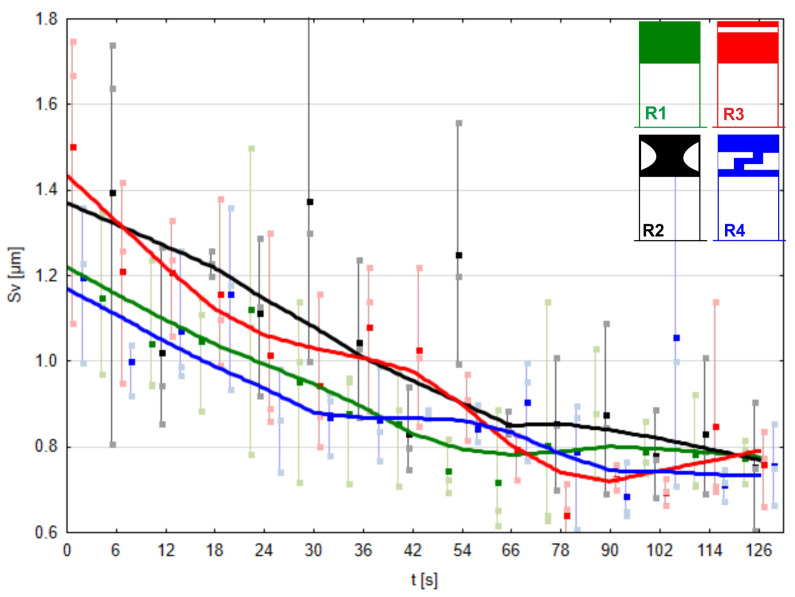
Maximum height of valleys on the machined surface as a function of processing time using the conventional pressure roller R1 and prototype rollers R2, R3, and R4.

The solid lines in Figure 15, Figure 16, Figure 17 and Figure 18 represent the Locally Weighted Scatterplot Smoothing (LOWESS). It is a data smoothing technique used in Statistica 13 software that involves fitting local regression models to data points. As a result, it produces a smooth curve that tends to reveal local trends in the data without introducing large oscillations. LOWESS fitting is used for trend analysis in data, eliminating noise and revealing more subtle patterns. Analyzing the Sa parameter for surface roughness evaluation, it can be confidently stated that pressure roller R3 removes surface irregularities most rapidly up to the 78 s mark of processing (Figure 15).

By the 78th second of processing, two pressure rollers, R3 and R4, provided the same quality of machined surface, with the Sa parameter measuring 0.095 μm, despite variations in surface quality before micromachining. The pressure roller R2 yielded the worst results at the 78 s mark of processing, although surface improvement occurred throughout the remainder of the machining process. Regarding the conventional roller R1 and prototypes R3 and R4, no surface improvement was observed after 78 s of processing, indicating that the maximum surface quality was achieved by this time. However, when analyzing the machining effects of the roller R2, surface improvement continued steadily after 78 s. The best results were achieved with pressure roller R4. 

The fact that after 78 s of processing with a 15-micrometer abrasive film changes in the Sa parameter occur much slower, indicating that the machined surface improves only slightly, is due to reaching the almost achieved maximum smoothing capacity of the tool, and further processing with such a tool is economically unjustified. This signals the need to switch to a tool with a lower grit size, as microfinishing is a sequential process. However, considering that our focus was on studying pressure rollers, the process utilized a 15-micrometer nominal size abrasive film.

Very promising results regarding surface roughness were obtained by analyzing the Sp parameter, which stands for the surface peak height (Figure 16). Surface peaks play a crucial role in the subsequent operational properties of the surface. The most spectacular results were achieved with the microfinishing process utilizing the prototype pressure roller R3, where surface peaks were removed most rapidly up to the 78 s mark of the micromachining process. Additionally, at the end of the finishing process, the best surface quality was achieved, with the Sp parameter measuring 0.1 μm less than the values of this parameter for surfaces finished using the other three rollers. When analyzing the removal of surface peaks, very similar effectiveness was observed for the conventional roller R1 and the most compliant rollers, R4 and R2. 

Analyzing the parameter for surface roughness assessment, Sz (Figure 17), which represents the surface height, also appears very favorable when using roller R3. This roller removed surface irregularities most rapidly up to the 90 s mark of the process, and we still achieved the best results at the end of the process. A similar rate of surface irregularity removal can be observed when using pressure roller R2. The effects obtained with the conventional roller R1 and the most compliant roller, R4, analyzing the Sz parameter of the processed surfaces, can be considered similar, both in terms of material removal speed and the achieved effects. This may also have a significant ecological aspect because producing roller R4 requires approximately one-third less elastomer.

**Figure 19:**
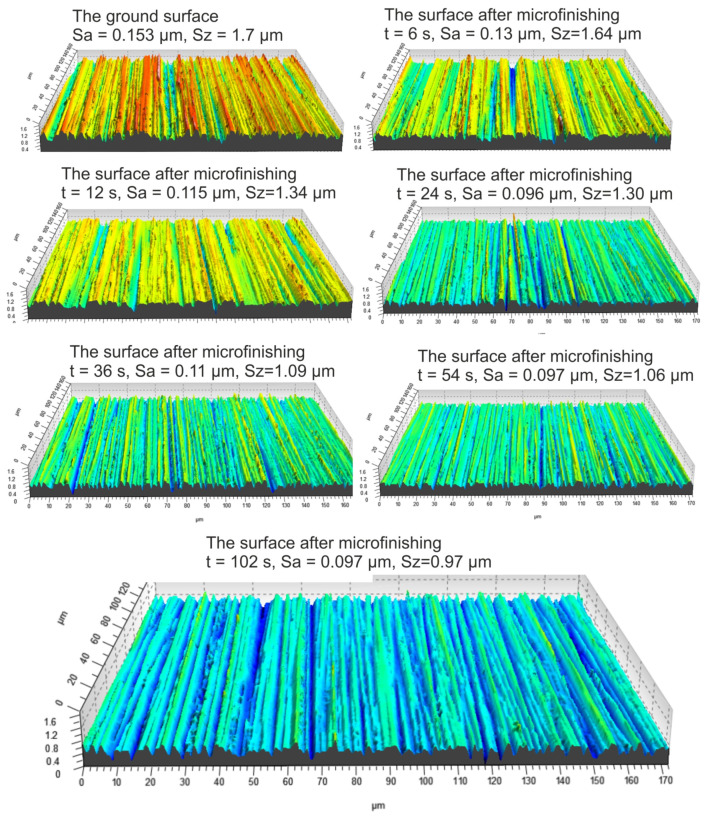
Images of the surface after different durations of micromachining with 15 IMFF abrasive film using pressure roller R4.

Analyzing the processed surfaces in terms of the Sv parameter (Figure 18), one can conclude that this parameter does not differentiate between surfaces microfinished using different pressure rollers. Images of the surface topography finished in a 3D layout are presented in Figure 19.

## 4. Summary and Conclusions

This article presents a comprehensive investigation into the pressure rollers utilized in the microfinishing process, encompassing their design, experimental properties, compliance, and finite element simulation. The research involved the design and fabrication of prototype pressure rollers with unconventional elastomer configurations, followed by experimental analysis to determine their compliance and surface contact area. Subsequently, finite element simulations were conducted to analyze stress and material displacement in the pressure rollers under loading conditions. Additionally, this article discusses the validation of the applied constitutive material model through experimental analysis of pressure roller deflection against a flat surface, highlighting the importance of selecting an appropriate material model for accurate simulation studies. Furthermore, this study includes insights into the contact zones of the simulated rollers, providing valuable information on how the contact behavior varies with deflection.

- The applied constitutive material model, utilizing the Blatz-Ko equation to describe the hyperelastic behavior of rubber, was validated through experimental analysis of pressure roller deflection against a flat surface. The agreement between the simulation and experimental results, reaching an overall level of 99.57%, confirms the suitability of the material model for further simulation studies. This validation underscores the importance of selecting an appropriate material model in accurately capturing the elastomer’s behavior under loading conditions, providing confidence in the reliability of subsequent finite element simulations in predicting pressure roller performance and deformation characteristics during microfinishing processes.
- The finite element simulations provided insights into the contact zones of the simulated rollers, varying with the deflection arrow. The analysis revealed distinct patterns of contact, with different pressure distributions and stress concentrations across the contact surfaces. As the deflection increased, the contact area widened, resulting in higher normal force values. Notably, prototype roller R4 exhibited the widest contact zone and the highest compliance, suggesting better adaptation to the machined surface’s curvature. Conversely, the conventional roller R1 demonstrated more localized pressure distribution, indicating a less conforming contact with the workpiece. These findings emphasize the significance of considering deflection-induced variations in contact behavior when designing pressure rollers for microfinishing applications.
- The quality of the obtained surfaces, particularly regarding surface peaks, plays a crucial role in determining the effectiveness of the microfinishing process. The investigation conducted in this study provides valuable insights into the performance of different pressure rollers in removing surface irregularities. Roller R3 exhibited the highest efficacy in removing surface peaks, indicating its potential to achieve superior surface finishes.

## Figures and Tables

**Table 1:** Material constants.

Workpiece Material (Steel)	
Density	7.85 × 10^−6^ kg mm^−3^
Coefficient of Thermal Expansion	1.2 × 10^−5^ C^−1^
Specific Heat	4.34 × 10^5^ mJ kg^−1^ C^−1^
Thermal Conductivity	6.05 × 10^−2^ W mm^−1^ C^−1^
Resistivity	1.7 × 10^−4^ ohm mm
Compressive Yield Strength	250 MPa
Tensile Yield Strength	250 MPa
Tensile Ultimate Strength	460 MPa
Reference Temperature	22 °C
** Blatz-Ko constitutive model (elastomer)**	
Density	2 × 10^−6^ kg mm^−3^
Initial Shear Modulus Mu	6.5 × 10^−2^ MPa

**Table 2:** Machining conditions for the experiments.

Workpiece Material	Chrome Steel (41Cr4)
Pressure roll hardness	80°Sh
Pressure force	60 N
Tool speed	160 mm/min
Workpiece speed	35 m/min

## Data Availability

Data are contained within the article.
